# Cervical Length Measurement for the Prediction of Preterm Birth in Symptomatic Women with a Twin Pregnancy: A Systematic Review and Meta-Analysis

**DOI:** 10.1155/2013/125897

**Published:** 2013-05-13

**Authors:** S. M. S. Liem, L. van de Mheen, D. J. Bekedam, M. G. van Pampus, B. C. Opmeer, A. C. Lim, B. W. J. Mol

**Affiliations:** ^1^Department of Obstetrics and Gynecology, Academic Medical Center, P.O. Box 22770, 1100 DE Amsterdam, The Netherlands; ^2^Department of Obstetrics and Gynecology, Onze Lieve Vrouwe Gasthuis, P.O. Box 95500, Amsterdam, The Netherlands; ^3^Clinical Research Unit, Academic Medical Center, P.O. Box 22770, Amsterdam, The Netherlands

## Abstract

*Objective*. The aim of this study was to assess whether cervical length measurement (CL) could predict preterm birth (PTB) in symptomatic women with a twin pregnancy. *Methods*. We searched MEDLINE and EMBASE to identify studies investigating the accuracy of CL measurement in predicting PTB in symptomatic women with a twin pregnancy. We extracted data to construct two-by-two tables and used bivariate meta-analysis to generate point estimates of sensitivity and specificity. *Results*. Five studies (*N* = 226) were included. Variation in definition of PTB and cut-off points for CL was strong. One study investigated delivery within seven days, demonstrating a sensitivity of 1.0 (95% CI: 0.83–1.0) and a specificity of 0.31 (95% CI 0.2–0.43) for a CL cutoff at 25 mm. Three studies reported on predicting PTB < 37 weeks at a CL cutoff of 30 mm, with sROC point estimates of 0.76 (95% CI: 0.66 to 0.84) and 0.37 (95% CI: 0.21 to 0.56) for sensitivity and specificity, respectively. For preterm birth <34 weeks, no pooled estimates could be estimated since only 2 studies with large heterogeneity were identified. *Conclusions*. There is limited evidence on the accuracy of cervical length measurement testing the prediction of preterm birth in symptomatic women with a twin pregnancy, especially on the most important outcome, that is, delivery within 7 days.

## 1. Introduction

Twin pregnancies are related to a significant higher rate of perinatal morbidity and mortality compared to singleton pregnancies [1, 2]. Preterm birth is the major contributing factor to this problem. In The Netherlands, approximately 50% of women with a multiple pregnancy deliver before 37 weeks of gestation, of whom 9% even prior to 32 weeks [3]. In the United States, these rates are 60% and 12%, respectively. In comparison, among women with a singleton pregnancy, 6%–10% delivers before 37 weeks' gestation and 1% prior to 32 weeks [3, 4].

Efforts in reducing the risk of preterm birth in twins have until now been unsuccessful. 

Excessive shortening of the cervical length in the second trimester in twin pregnancies is one of the best predictors of preterm delivery [5–7]. Different studies showed that ultrasonographic cervical length is superior to digital assessment. It may safely provide precise, objective, and reproducible measurement of cervical length [8, 9]. Introduction of transvaginal ultrasonographic measurement of the cervical length reduced the hospital stay from 18 to 10 days in singleton pregnancies, while the number of preterm births remained stable [10].

Several reviews show that transvaginal cervical sonography identifies women at increased risk of spontaneous preterm birth, although there usually is a wide variation amongst studies in gestational age at testing, definition of threshold of abnormality, and definition of reference standard [11–15]. These studies mainly describe the use of CL in asymptomatic women. In symptomatic women, however, the clinical relevance is to distinguish between women who will truly deliver and those who will not. Correct identification of these women might be effective in reducing perinatal morbidity and mortality by providing needed interventions such as tocolysis, antenatal corticosteroid administration, and transfer to a tertiary care center in time. Furthermore, the mechanism of preterm birth in women with a twin pregnancy is unclear and is likely to differ from women with a singleton pregnancy. It seems that relative overdistension of the uterus is the most common aetiology causing PTB in twin pregnancies [16]. Given different incidence, of PTB and different mechanisms leading to PTB, we believe that transvaginal sonographic cervical length measurement in twin pregnancy to predict PTB should be evaluated separately. 

The aim of this review was to access the accuracy of transvaginal sonographic CL in predicting preterm birth in women with a twin pregnancy and symptoms of preterm birth. We conducted a meta-analysis using bivariate regression analysis, accounting for correlation between sensitivity and specificity.

## 2. Methods

### 2.1. Search Strategy

We searched the electronic databases of MEDLINE (US National Library of Medicine, Bethesda, MD, USA) and EMBASE (Elsevier, Amsterdam, The Netherlands) from inception to July 2012. The search strategy included MeSH or key terms related to cervical length, multiple pregnancy, and preterm birth. We checked reference lists to identify articles not found by electronic searches. We identified studies that reported on cervical length for the prediction of preterm birth in women with a twin pregnancy and suspected preterm birth. We did not apply any restrictions concerning study design.

### 2.2. Study Selection

The initially identified articles were screened by two independent reviewers (S. Liem and L. van de Mheen) on title and abstract to determine their appropriateness for inclusion. The studies should have preterm birth as their primary or secondary outcome and should be reporting on sonographically measured cervical length in women with a multiple pregnancy and threatened preterm birth. Symptoms of preterm birth were defined as the occurrence of uterine contractions, cervical effacement, dilatation, or change in consistency. If studies could not be excluded based on their abstract or title, a full manuscript was obtained. We did not apply any language restrictions. When an article was written in another language than Dutch or English, it was translated by a colleague with expertise in this language. For each study, we constructed a two-by-two table cross-classifying cervical length and gestational age. If information available from the publications was not sufficient, the primary authors were contacted. In case of any disagreements about study inclusion, the two reviewers had a discussion. If consensus could not be reached, a third reviewer (B. Mol) determined whether the study should be included. 

### 2.3. Data Extraction

The two reviewers abstracted the data separately. We designed a data abstraction form on study characteristics, test characteristics, definition of outcome, study quality, and participant characteristics. Methodological quality of included studies was determined by using an adapted version of QUADAS by both reviewers independently [17]. The study had a representative spectrum of patients when pregnant women were consecutively selected in a prospective way. The description of the test was classified as adequate if the study at least described if the bladder was empty and whether funnelling was included or excluded in the cervical length. When it is clearly stated that the treating clinician was blinded for the outcome of the CL results, the study was considered blinded. If this was not clear, this item was scored as “no.” If the study withdrawals were defined or a flow diagram was reported, this item was scored with “yes.” We studied two end points. First, we evaluated the predictive capacity of cervical length to predict preterm delivery, defined as delivery before 34 or 37 weeks. Second, we evaluated whether cervical length could predict delivery within 7 days from inclusion.

### 2.4. Data Synthesis and Statistical Analysis

For each study, two-by-two classification tables were reconstructed, based on reported data on true and false positive and true and false negative test results. If studies reported on the accuracy for more than one threshold for cervical length or for more than one definition of preterm birth, multiple two-by-two tables were reconstructed. 

Sensitivity and specificity with 95% confidence intervals (CIs) were calculated for each study and for reported cut-off values. To explore heterogeneity of the results, we created forest plots of sensitivity and specificity and plotted their combined results in summary receiver operating characteristics (sROC) space. As estimates may be based on different positivity thresholds (explicitly defined or implicitly present), part of the observed heterogeneity could reflect a shift along an underlying sROC curve.

Pooled estimates of sensitivity and specificity were estimated simultaneously using the bivariate model [18]. Summary ROC curves were pooled-estimated from the model parameters and plotted with the original data points in the sROC space [19].

If we estimated accuracy for a single combination of threshold values for cervical length and preterm birth, this estimate would be based on only a limited number of studies. Furthermore, it is not clear which is the appropriate threshold for either definition. In order to evaluate accuracy measures over the whole range of possible thresholds, however, we did not limit our analysis to a single threshold value, but estimated accuracy measures for all reported threshold values by assuming that the shift in accuracy (higher sensitivity and lower specificity) due to different thresholds is accounted for by the correlation term in the bivariate model. Pooled estimates were based on averages of repeated stratified bootstrap samples to account for multiple accuracy points reported by the same study. To avoid results being biased towards studies reporting on many different thresholds, we estimated each model in 100 stratified bootstrap samples, in which only one accuracy estimate from each study was randomly selected (stratified by study). For each parameter, the average overall estimate from 100 bootstrap samples is reported, and all studies equally contributed to this estimate.

Therefore, three types of analysis were performed. The first type included all reported accuracy estimates, irrespective of threshold values for cervical length and preterm birth or gestational age at which cervical length was measured, and resulted in a sROC curve; the second type included only accuracy pooled estimates for two different cervical length thresholds (25 and 30 mm). For the third type, we analyzed preterm birth <37 weeks and a CL cutoff of 30 mm; we reported an sROC point estimate and 95% confidence ellipse.

## 3. Results

**Figure 1:**
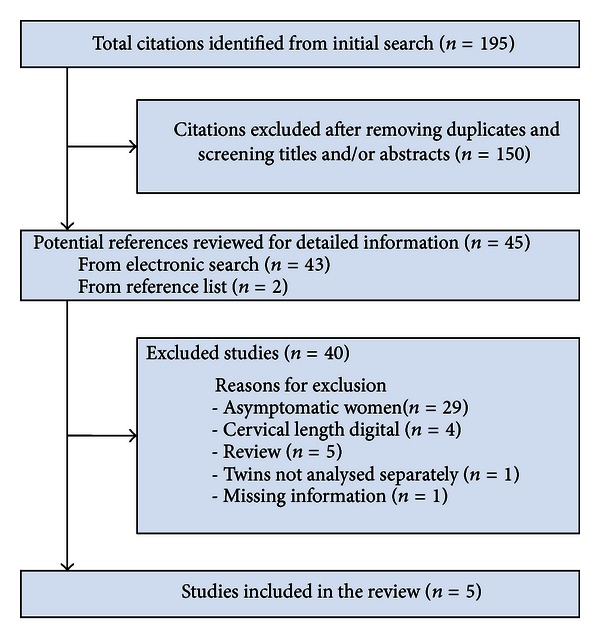
Flowchart.

The search revealed 195 potentially eligible abstracts of which 45 were considered relevant after reading title and abstract. We excluded 40 of these 45 studies for different reasons: reporting on asymptomatic women (*N* = 29), digital assessment of cervical length (*N* = 4), systematic review (*N* = 5), no separate analysis for twins (*N* = 1), and lack of original data (*N* = 1) (Figure 1). 

Main characteristics of included studies are summarized in Table 1. All five identified studies were prospective cohort studies, with a sample size ranging from 21 to 87 women.

Only one study reported on delivery within 7 days. The study included women with painful and regular uterine contractions at 24–36 weeks of gestation who were included, while women who had >3 cm cervical dilatation, ruptured membranes, or cervical cerclage were excluded. Delivery within 7 days, which occurred in 19 (22%) of the 87 patients, was inversely related to cervical length. This occurred in 80% of women with a CL between 1 and 5 mm, 46% of women with a CL between 6 and 10 mm, 29% with a CL between 11 and 15 mm, 21% of women with a CL between CL 16 and 20 mm, 7% of women with a CL between 21 and 25 mm, while nobody delivered within 7 days if the CL was >25 mm. The sensitivity and specificity for delivery within seven days at a CL cutoff of 25 mm were 1.0 (95% CI: 0.83–1.0) and 0.31 (95% CI: 0.2–0.43), respectively [20].

Crane et al. [5] included women with singleton and twin pregnancies presenting with regular uterine contractions with dilatation or cervical change between 23 and 33 weeks of gestation. Women with advanced labor, cervical dilation >3 cm, vaginal bleeding, placenta previa, ruptured membranes, cervical cerclage, and a stillbirth were excluded. Twin pregnancies (*N* = 26) were analysed separately for the outcome PTB < 37 and <34 weeks and CL cutoff <30 mm. Sensitivity and specificity for PTB < 37 weeks and CL cutoff <30 mm were 0.75 (95% CI: 0.51–0.90) and 0.30 (95% CI: 0.11–0.60). PTB < 34 weeks and CL cutoff <30 mm showed sensitivity and specificity of 1.0 (95% CI: 0.57–1) and 0.57 (95% CI: 0.37–0.76). Gonzalez et al. [21] investigated singleton and twin pregnancies presenting with regular uterine contractions or cervical change <34 weeks of gestation. Women with a cervical cerclage, ruptured membranes, maternal pathology, or fetal contraindication to continue the pregnancy were excluded. Twin pregnancies (*N* = 66) were analysed separately for PTB < 37 weeks and CL cutoff <30 mm (sensitivity 0.73 (95% CI: 0.61–0.83); specificity 0.33 (95% CI: 0.09–0.7)), PTB < 34 weeks and CL cutoff <30 mm (sensitivity 0.75 (95% CI: 0.57–0.87); specificity 0.29 (95% CI: 0.17–0.45)), and PTB < 34 weeks and CL cutoff <20 mm (sensitivity 0.46 (95% CI: 0.29–0.64); specificity 0.68 (95% CI: 0.53–0.81)). 

Vendittelli and Voluménie [22] included women with a singleton or twin pregnancy and regular uterine contraction or cervical changes between 18 and 36 weeks of gestation. Women with cervical dilatation >3 cm, ruptured membranes, cervical cerclage, vaginal bleeding, placenta previa, stillbirth, fetal malformation, and delivery within 24 hours after admission were excluded. Twenty-six women with a twin pregnancy were analysed separately. Sensitivity and specificity for PTB < 37 weeks and CL cutoff <30 mm were 0.87 (95% CI: 0.62–0.96) and 0.46 (95% CI: 0.21–0.72), respectively.

Yoshizato et al. [23] investigated women with a twin pregnancy and regular uterine contractions or cervical ripening. Women with a potential medical background leading to PTB, such as cone biopsy, uterine anomalies, maternal complication like hypertension or diabetes, history of miscarriage or PTB between 16 and 32 weeks, vaginal bleeding, or twin-to-twin syndrome were excluded. In all cases (*N* = 21) tocolysis was required starting at 25–33 weeks of gestation and continued till 35 weeks or delivery. PTB < 37 weeks and CL cutoff <25 mm showed sensitivity of 1.0 (95% CI: 0.76–1.0) and specificity of 0.11 (95% CI: 0.02–0.44).

Table 2 demonstrates for each individual study the observed sensitivity and specificity with 95% CI, positive predictive value, negative predictive value, and likelihood ratios of positive and negative test results. Table 1 summarizes the quality of the included studies. All studies had a representative spectrum of patients and an adequate description of the test procedure. In one study, the clinician was blinded for the CL measurement, whereas two studies reported on study withdrawals.

**Figure 2:**
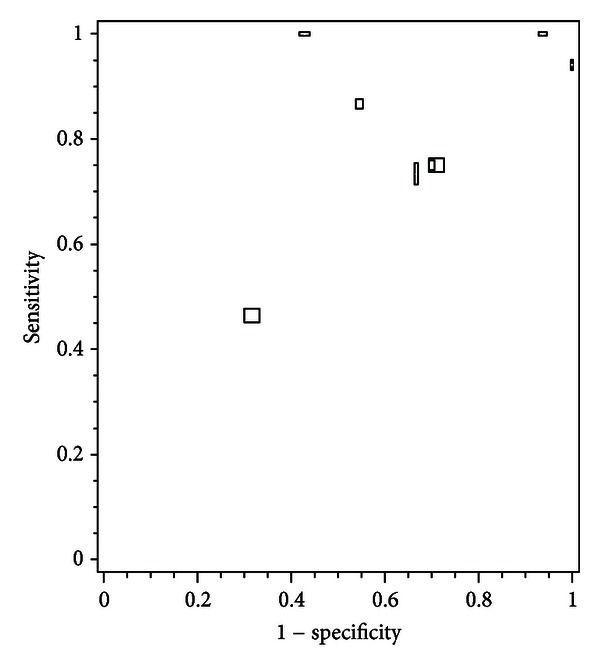
Summary receiver operating characteristics plot for observed accuracy for all individual studies. (Observed accuracy for studies reporting on different cervical length thresholds and definitions of preterm birth were plotted separately).

Figure 2 shows a summary ROC plot for the observed sensitivity and specificity for the prediction of spontaneous preterm birth in symptomatic women with a twin pregnancy for all individual studies. We planned a meta-analysis for the reported accuracy estimates, irrespective of threshold values for cervical length and preterm birth or gestational age at which cervical length was measured. Primary studies showed large heterogeneity in CL cutoffs, definitions of PTB, gestation age at testing, and inclusion criteria, thus complicating pooling of the results in meta-analysis.

For the subgroup analyses for women with a CL cutoff below 25 mm and 30 mm, we explored the data from the primary studies. Only two studies reported on PTB and CL cutoff <25 mm. Gonzalez et al. [21] reported higher sensitivity and specificity at a CL cutoff of 30 mm compared to 20 mm. Since these results are unclear, we tried to contact the authors of primary studies. We were unable to obtain information that could clarify our uncertainties from the authors. Most authors did not response or replied that the data was no longer available. Therefore, we decided not to conduct this analysis.

**Figure 3:**
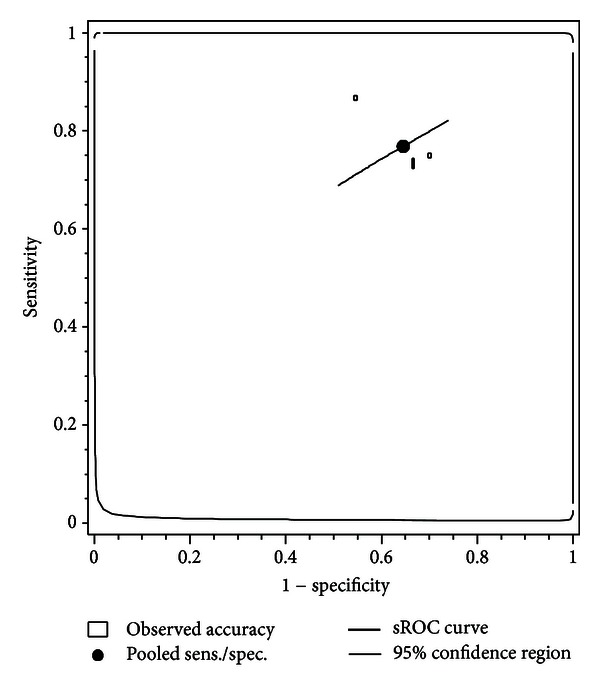
Summary receiver operating characteristics curve with 95% confidence interval for preterm birth <37 weeks and cervical length cutoff <30 mm.

Three studies specifically reported on CL cutoffs of 30 mm and PTB < 37 weeks [5, 21, 22].  Figure 3 shows the sROC curve with 95% CIs for predicting preterm birth <37 weeks for a CL cutoff of 30 mm with a sensitivity of 0.76 (95% CI: 0.66 to 0.84) and specificity of 0.37 (95% CI: 0.21 to 0.56). For preterm birth <34 weeks, no pooled estimates could be estimated since we only identified 2 studies reporting on this cutoff showing large heterogeneity, but the accuracy estimates reported by the individual studies suggest that CL measurement had poor predictive accuracy. 

## 4. Discussion 

After an extensive search, we only identified 5 studies with relative small sample sizes that could be included in this review on the predictive capacity of cervical length measurement for preterm birth in symptomatic women with a twin pregnancy. Variation in definition of PTB, cut-off points for CL, and gestational age was large. It is remarkable that a test used in daily obstetric care is evaluated in such a low number of studies. Four of these studies focused on preterm birth before 34 or 37 weeks. We believe that these outcomes are less relevant since, in view of the clinical problem of symptomatic PTB, it is important to distinguish between symptomatic women who will deliver within short time and women who can be sent home safely without additional treatment. 

A meta-analysis in 1781 women with a singleton pregnancy presenting with threatened preterm birth showed that a CL < 15 mm could predict 60% of preterm births within one week [24]. In our opinion, tests to predict PTB in symptomatic women are most relevant when they predict PTB within a short interval rather than delivery before 34 or 37 weeks. Correct identification of these women might be effective in reducing perinatal morbidity and mortality by providing needed interventions such as tocolysis, antenatal corticosteroid administration, and transfer to a tertiary care center in time. Therefore, studies should focus on delivery within 48 hours or within 7 days as the most important study outcomes. We identified only one study that reported on twin pregnancies with symptoms of PTB and delivery within 7 days. This study showed sensitivity and specificity of 1.0 (95% CI: 0.83–1.00) and 0.31 (95% CI: 0.2–0.43) for delivery within seven days and a CL cutoff <25 mm [20].

We planned a subgroup analysis for women with a CL cutoff below 25 mm and 30 mm; therefore, we explored our primary data. Gonzalez et al. reported higher sensitivity and specificity for a CL cutoff below 30 mm compared to 20 mm, and these results seem contradictory. We contacted the author to obtain additional information on the individual CL measurements, but unfortunately we did not obtain the needed information. Since there is limited evidence on CL measurement in women with a twin pregnancy and symptoms of PTB, we decided not to completely exclude this study from further analysis. Also the other data from this study is in accordance with the identified primary studies. 

Three studies specifically reported on predicting PTB < 37 weeks and a CL cutoff of 30 mm [5, 21, 22]. The sROC point estimates for sensitivity and specificity were 0.76 (95% CI: 0.66 to 0.84) and 0.37 (95% CI: 0.21 to 0.56), respectively.

For screening tests, a substantially higher sensitivity is required, because with a negative test result (CL >30 mm) preterm birth needs to be ruled out.

We performed a rigorous systematic review of predictive test accuracy. The extensive search without language restriction, the systematically assessment for quality, and techniques recommended for meta-analysis of diagnostic and predictive tests and investigation for sources of heterogeneity all contributed to the strength of our review. As expected, this review is limited by the quality of the included studies. Also, there was substantial heterogeneity in study characteristics among individual studies in which meta-analyses were performed. In addition to the limited predictive accuracy, there is substantial statistical uncertainty due to the small number and sample sizes of the included studies. Furthermore, the symptoms of preterm birth were different between the women and studies. Women with regular painful contractions and cervical change might be at greater risk of preterm delivery than women with regular contractions only. In four studies, the clinician was not blinded for the CL measurement, and this may have led to information bias. Nevertheless, we did not find important differences between the studies where the clinician was not blinded for the CL measurement versus the study where CL was unknown to the treating clinician.

Our meta-analyses provided limited evidence to recommend the use of CL measurement to predict PTB in symptomatic women with a multiple pregnancy. One should realize that in women with a twin pregnancy midtrimester shortening of the CL is already a common phenomenon. Thus, one should be careful in using cut-off values for cervical length in symptomatic women. As demonstrated before and confirmed in this study, such a short cervix is associated with preterm delivery, but the evidence on prediction within 7 days is rarely assessed. Probably, cutoffs for prediction of symptomatic women should be made with care. 

In summary, this meta-analysis shows that sensitivity and specificity of CL measurement for predicting preterm birth in symptomatic women with a twin pregnancy are relatively low. In view of the limited evidence on the accuracy of cervical length measurement to predict preterm birth in symptomatic women with a twin pregnancy and in view of the poor predictive capacity, we are unable to formulate recommendations for the routine use of CL measurement in symptomatic women with a multiple pregnancy to predict PTB. We believe that a test to predict PTB in symptomatic women with a multiple pregnancy is only clinically relevant when it predicts delivery within 48 hours or 7 days rather than PTB < 34 or <37 weeks. Future research on evaluating the effectiveness of CL measurement in predicting preterm birth in symptomatic women with a multiple pregnancy should focus on delivery within 48 hours or 7 days. 

## Figures and Tables

**Table 1:** Study characteristics.

Study	Inclusion criteria	Exclusion criteria	*n*	Gestational age at testing (wk)	CL at inclusion	CL cutoff (mm)	Outcome	Design	Representative spectrum of patients	Adequate description of test procedure	Practitioner blinded to measurement results	Reporting on study withdrawals
Fuchs	Twins, regular and painful uterine contractions	Cervical cerclage, ruptured membranes, and dilatation >3 cm	87	24–36	20 mm (1–45) Median (IQR)	25	Delivery within 7 days	Prospective cohort	Yes	Yes	No	Yes

Crane	Twins, regular uterine contraction with cervical dilatation, effacement, and/or change in consistency	Cervical cerclage, placenta previa, ruptured membranes, vaginal bleeding, dilatation >3 cm, and stillbirth	26	23–33	25 mm (14.0) Mean (SD)	30	Preterm birth <34 and <37 weeks	Prospective cohort	Yes	Yes	No	No

Gonzalez	Twins, regular uterine contraction with cervical changes (effacement of at least 50% or dilatation of at least 1 finger)	Cervical cerclage, maternal or fetal contraindication to continue pregnancy	66	<34	Not reported	20, 30	Preterm birth <34 and <37 weeks	Prospective cohort	Yes	Yes	No	No

Venditelli	Twins, regular uterine contraction, and/or cervical effacement or dilatation	Cerclage, fetal anomalies, placenta previa, ruptured membranes, vaginal blood loss, dilatation >3 cm, stillbirth, and delivery <24 weeks after admission	26	18–36	Not reported	30	Preterm birth <37 weeks	Prospective cohort	Yes	Yes	Yes	Yes

Yoshizato	Twins, regular uterine contraction with cervical dilatation, effacement, and/or change in consistency	TTTS, uterine anomalies, conization, vaginal blood loss, PE, hypertension, diabetes, and history of PTB	21	16–35	22 mm (5.5) Mean (SD)	25	Preterm birth <34 and <37 weeks	Prospective cohort	Yes	Yes	No	No

**Table 2:** Observed sensitivity and specificity for each study with 95% CI.

Study	*N*	Cut-off CL	Cut-off PTB	TP	FP	FN	TN	Sens.	95% CI	Spec.	95% CI	PPV	NPV	LR+	LR−
Fuchs	87	25	<7 days	19	47	0	21	1.0	0.83–1.0	0.31	0.31–0.43	0.29	1.0	1.5	0
Crane	26	30	34	5	9	0	12	1.0	0.57–1.0	0.57	0.37–0.76	0.36	1.0	2.3	0
Crane	26	30	37	12	7	4	3	0.75	0.51–0.90	0.30	0.11–0.60	0.63	0.43	1.1	0.83
Gonzalez	66	20	34	13	12	15	26	0.46	0.29–0.64	0.68	0.53–0.81	0.52	0.63	1.5	0.78
Gonzalez	66	30	34	21	27	7	11	0.75	0.57–0.87	0.29	0.17–0.45	0.44	0.61	1.1	0.86
Gonzalez	66	30	37	44	4	16	2	0.73	0.61–0.83	0.33	0.09–0.70	0.92	0.11	1.1	0.80
Venditelli	26	30	37	13	6	2	5	0.87	0.62–0.96	0.46	0.21–0.72	0.68	0.71	1.6	0.29
Yoshizato	21	25	37	12	8	0	1	1.0	0.76–1.0	0.11	0.02–0.55	0.60	1.0	1.1	0

TP: true positive; FP: false positive; FN: false negative; TN: true negative; PPV: positive predictive value; NPV: negative predictive value; LR+: likelihood ratio positive; LR−: likelihood ratio negative.
